# Single-Dose Versus Multiple-Dose GnRH Agonist for Luteal-Phase Support in Women Undergoing IVF/ICSI Cycles: A Network Meta-Analysis of Randomized Controlled Trials

**DOI:** 10.3389/fendo.2022.802688

**Published:** 2022-03-31

**Authors:** Yang Liu, Yanzhi Wu, Zhengmei Pan, Fangjie Jiang, Youhui Lu, Yushi Meng

**Affiliations:** Department of Reproduction, The Second Affiliated Hospital of Kunming Medical University, Kunming, China

**Keywords:** *in vitro* fertilization, intracytoplasmic sperm injection, gonadotropin-releasing hormone agonist, luteal-phase support, network meta-analysis

## Abstract

**Background:**

Although gonadotropin-releasing hormone (GnRH) agonist has been introduced as a beneficial luteal phase support (LPS), the optimal strategy of GnRH agonist remains unclear. This network meta-analysis was therefore performed to determine the comparative efficacy and safety of multiple-dose versus single-dose GnRH agonist protocol for LPS in patients undergoing IVF/ICSI cycles.

**Methods:**

We searched relevant studies in PubMed, Embase and the Cochrane Registry of Controlled Trials (CENTRAL) from their inception util to September 2021. Live birth, clinical pregnancy rate, multiple pregnancy rate, and clinical abortion rate was evaluated. Pairwise and network meta-analysis were conducted using RevMan and ADDIS based on random-effects model, respectively. Moreover, the prioritization of protocols based on ranking probabilities for different outcomes were performed.

**Results:**

Sixteen RCTs met our eligibility criteria. Pairwise meta-analysis showed that multiple-dose protocol of GnRH agonist was effective for increasing live birth rate (OR 1.80, 95% CI 1.15 to 2.83, *p*=0.01) and clinical pregnancy rate (OR 1.89, 95% CI 1.01 to 3.56, *p*=0.05) as well as decreasing clinical abortion rate (OR 0.55, 95% CI 0.34 to 0.90, *p*=0.02). Meanwhile, single-dose protocol of GnRH agonist was effective for increasing clinical pregnancy rate (OR 1.45, 95% CI 1.11 to 1.89, *p*=0.007) and multiple pregnancy rate (OR 2.55, 95% CI 1.12 to 5.78, *p*=0.03). However, network meta-analysis only confirmed that multiple-dose protocol of GnRH agonist was the best efficacious strategy for live birth rate (OR 2.04, 95% CrI 1.19 to 3.93) and clinical pregnancy rate (OR 2.10, 95% CrI 1.26 to 3.54).

**Conclusion:**

Based on the results of NMA, multiple-dose protocol may be the optimal strategy for patients undergoing IVF/ICSI cycles owing to its advantage in increasing live birth and clinical pregnancy rate. Moreover, single-dose protocol may be the optimal strategy for improving multiple pregnancy rate. However, with the limitations, more RCTs are required to confirm our findings.

## 1 Introduction

*In vitro* fertilization (IVF)/intracytoplasmic sperm injection (ICSI) has been extensively accepted for fertility aid among couples with infertility, and more than 1 million cycles were actually reported every year around the world (1). However, patients receiving IVF/ICSI cycles commonly suffered from luteal-phase deficiency (LPD) due to the use of controlled ovarian stimulation (COS) based on gonadotrophin-releasing hormone agonist (GnRH-a) or antagonist protocols (2–4). It’s noted that LPD was linked to several adverse pregnancy outcomes, such as a relatively lower embryo implantation rate, clinical pregnancy rate and live birth rate (5). Therefore, it’s vitally important to provide exogenous luteal-phase support (LPS) for paying compensation to the progesterone levels (6). Actually, a number of LPS protocols have been investigated, such as estradiol, progesterone, human chorionic gonadotropin (hCG), gonadotropin-releasing hormone (GnRH) agonists or combinations of these types (7, 8).

Among available exogenous LPS protocols, progesterone and hCG were widely used in clinical practice. However, compared with progesterone, hCG was linked to increased risk of ovarian hyperstimulation syndrome (5, 9). From the perspective of safety, progesterone should be preferentially selected. Nevertheless, the optimal administration route remains unclear (10, 11). Beyond that, some other modalities are currently under investigation, such as estrogen, ascorbic acid, and acupuncture (12). It’s exciting that gonadotropin-releasing hormone (GnRH) agonist protocol recently introduced as a beneficial LPS (13, 14), and studies have indicated the positive role of the administration of a single-dose GnRH agonist protocol in IVF/ICSI cycles (2, 15–17).

However, in addition to single-dose administration, multiple-dose protocol of GnRH agonist as a LPS protocol has become more and more common (16). Up to now, the paucity of studies directly compared single-dose versus multiple-dose GnRH agonist protocol (18–20) although there were relatively numerous studies directly comparing single-dose (16) or multiple-dose (21–23) protocol with control protocols, respectively. As a result, optimal administration strategy of GnRH agonist remains debated. We therefore collected all available randomized controlled trials (RCTs) to conduct a network meta-analysis for the comparative efficacy and safety in single-dose versus multiple-dose GnRH agonist protocols among patients undergoing IVF/ICSI cycles. We also provided the hierarchies of the comparative live birth rate, clinical pregnancy rate, multiple pregnancy rate and clinical abortion rate on two protocols.

## 2 Materials and Methods

We performed the present network meta-analysis according to the preferred reporting items for systematic reviews and meta-analysis (PRISMA) for network meta-analysis (PRISMA-NMA) (24, 25) and the Cochrane handbook for reviewer of systematic review (26). We did not register the formal protocol in public platform.

### 2.1 Search Strategy

We developed the search strategy according to a previous meta-analysis (16). We firstly identified five distinctive keywords as follows: gonadotropin-releasing hormone agonist, single-dose, multiple-dose, fertilization *in vitro*, and intracytoplasmic sperm injections. Then, we identified the medical subject heading (MeSH) based on MeSH database, and further determined possible expressions of all keywords. An electronic literature search was independently performed by two reviewers in PubMed, Embase (based on Ovid) and the Cochrane Registry of Controlled Trials (CENTRAL) (based on Ovid) from their inception until to 30 September 2021. Detailed search strategy were summarized in Table S1.

### 2.2 Eligibility Criteria

According to previous meta-analysis (16), we included RCTs which assessed the comparative efficacy and safety of single-dose versus multiple-dose GnRH agonist protocols as LPS on IVF/ICSI outcomes in this network meta-analysis. The following exclusion criteria were imposed: (a) studies with ineligible design, such as summary, discuss theory, letters, case reports, comments, meta-analysis, review, and other types of research literature; (b) duplicate publications and data were unavailable to odds ratios (OR); (c) patients with egg donation and frozen embryo transfer.

### 2.3 Study Selection

Results retrieved were firstly imported into EndNote software for the removal of duplicate records and initial screening. Manual forwards and backwards reference searching were done on all included studies to identify additional relevant studies. Titles, abstracts, and full texts were examined independently by two reviewers. Any conflicting was resolved through discussion until the consensus was achieved.

### 2.4 Definition of Outcomes

We evaluated four outcomes in this network meta-analysis, including live birth rate, clinical pregnancy rate, multiple pregnancy rate and clinical abortion rate. We used the ongoing pregnancy rate as the surrogate of the live birth rate when the data was not available because of the difference between both two data can be ignored (27, 28). When studies reported on clinical pregnancy and ongoing pregnancy without miscarriage rates, the number of clinical abortion rate was seen as being equal to the difference between the number of clinical pregnancy rate and ongoing pregnancy rate.

### 2.5 Data Extraction

The following essential data were collected from each study, including the name of the first author, year of publication, sample size and country, details of ovarian stimulation protocol, details of LPS protocols, statistical findings and details of methodological quality. Two independent reviewers collected these data, and any conflicting between the two reviewers was settled by consensus principle.

### 2.6 Evidence Network

We displayed the current status of available evidence in terms of all outcomes through creating evidence network which was conducted using ADDIS software. In evidence network, solid line connecting two protocols indicated the presence of direct comparison and the numerical value marked in line indicated the number of eligible studies for each direct comparison.

### 2.7 Quality Assessment

Two independent reviewers used the Cochrane Risk of Bias tool (29) to assess the risk of bias from 6 aspects as follows: selection bias (random sequence generation and concealment of allocation), performance bias (blinding of investigators and participants), detection bias (blinding of outcome assessors), attrition bias (incomplete outcome data), reporting bias (selective reporting), and other sources of bias (e.g., inadequate sample size and funding bias). Each aspect was labeled as “low”, “unclear”, or “high” risk of bias according to the actual information. Consensus principle was utilized to address inconsistency between two reviewers.

### 2.8 Statistical Analysis

#### 2.8.1 Pairwise Meta-Analysis

We firstly used RevMan version 5.4 (Review Manager, the Cochrane Collaboration, 2020) to conduct pairwise meta-analysis. Heterogeneity across studies was assessed using the Cochrane Q test (30) and *I^2^* value (31). All outcomes which were analyzed in this network meta-analysis were categorical variables, and thus we used the odds ratio (OR) with corresponding 95% confidence interval (CI) to express pooled estimates of all outcomes.

#### 2.8.2 Network Meta-Analysis

We utilized the Aggregate Data Drug Information System (ADDIS) V.1.16.8 (Drugis, Groningen, NL) to conduct network meta-analysis (32). A Bayesian network meta-analysis was performed using a random effects model for integrating the direct and indirect evidence with the Markov Chain Monte Carlo (MCMC) method. The parameters for the network meta-analysis in the ADDIS were as follows: the number of chains, 4; tuning iterations, 20,000; simulation iterations, 50,000; thinning interval, 10; inference samples, 10,000; and variance scaling factor, 2.5 (33). The potential scale reduced factor (PSRF) was used to evaluate convergence (34, 35). Good convergence was achieved when PSRF was close to or equaled to 1, indicating high reliability of the conclusions of the consistency model analysis. Model convergence will be assessed by visual inspection of the trace plots and after considering the Gelman-Rubin statistic. Results from network meta-analysis were expressed as OR, accompanied with 95% credible interval (CrI). Before conducted quantitative synthesis, we firstly used split node method to examine the possibility of inconsistency between direct and indirect effects (36, 37). A consistency model was used when the P value >0.05 in the node-splitting analysis; otherwise, the inconsistency model was used. Finally, the relevant rank plots based on probabilities of intervention for the different endpoints were shown by ADDIS (38).

#### 2.8.3 Assessment of Publication Bias

We used funnel plot, which was created using RevMan software, to evaluate the robustness of pooled results when the accumulated numbers of eligible studies were more than 10 (39).

## 3 Results

### 3.1 Identification of Studies

**Figure 1:**
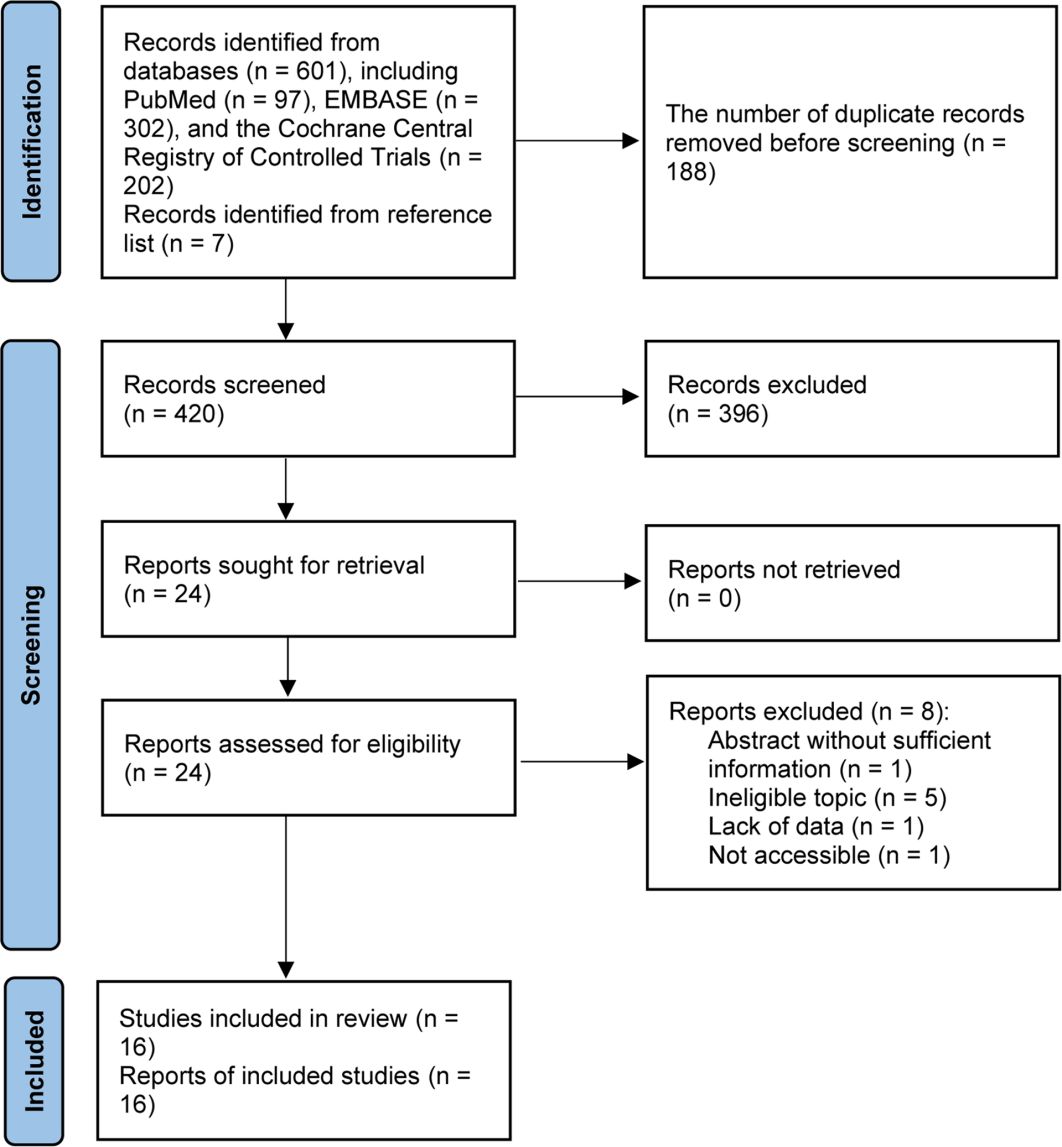
Flow chart for the literature search and study selection.

We identified 601 records from electronic databases. Total 420 studies were retained for initial screening based on the titles and abstracts after the removal of 188 duplicate records. Among them, 24 studies were retrieved for full-text evaluation after excluding 396 ineligible studies. Then, 9 unique studies (18, 19, 21, 40–45) were considered to meet selection criteria after excluding 8 ineligible studies due to conference abstract without sufficient data and ineligible topic. Additional 7 eligible studies (13, 22, 23, 46, 47) were retrieved from previous meta-analysis and reference lists, and then we included 16 eligible studies (13, 18, 19, 21–23, 40–49) in this network meta-analysis. Flow chart for the literature search and study selection was presented in **Figure 1**.

### 3.2 Characteristics of the Included Studies

**Figure 2:**
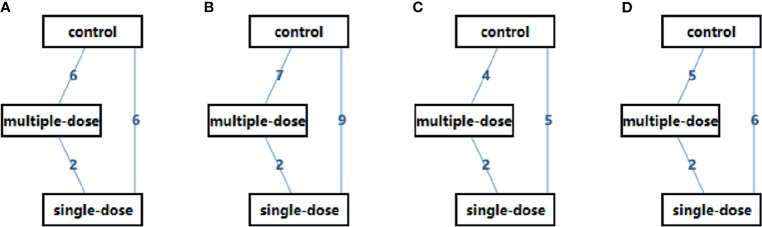
Evidence Network of the eligible studies. **(A)** live birth rate, **(B)** clinical pregnancy rate, **(C)** multiple pregnancy rate, and **(D)** clinical abortion rate. D, single-dose; MD, multiple-dose.

Among 16 eligible studies, majority of studies were published in Turkey (18, 40–42) and Iran (21, 43–45), and remaining studies were published in Spain (49), Denmark (48), Japan (13), India (22), Belgium (46), Jordan (47), and China (19), respectively. Eight studies (40–45, 48, 49) compared single-dose versus controls, six studies (13, 21–23, 46, 47) compared multiple-dose versus controls, one study (19) compared single-dose versus multiple-dose, and one study (18) simultaneously single-dose, multiple-dose, and control regime. **Table 1** shown the basic characteristics of each study, and **Figure 2** displayed the evidence network of each outcome.

**Table 1:** Characteristics of the included studies (n=16).

Author	Country	Sample size		Condition	Ovarian stimulation protocol	LPS protocol		Control	Other protocol	Day after ER
		Randomization	Final Analysis			SD protocol	MD protocol			
Tesarik, et al., (49)	Spain	300 *vs* 300	286 *vs* 286	ICSI	Long GnRH agonist protocol and GnRH antagonist protocol	0.1mg triptorelin 6 days after ICSI	n.a.	placebo	4mg E2 valerate, 400mg vaginal micronized progesterone, 250μg human recombinant hCG	3
Ata, et al., (41)	Turkey	285 *vs* 285	285 *vs* 285	ICSI	Long GnRH agonist protocol and r-FSH	0.1mg triptorelin 6 days after ICSI	n.a.	placebo	90mg vaginal progesterone gel	3
Ata, et al., (40)	Turkey	n.r.	38 *vs* 52	ICSI	Long GnRH agonist protocol and r-FSH	0.1mg triptorelin 6 days after ICSI	n.a.	placebo	90mg vaginal progesterone gel	3
Isik, et al., (42)	Turkey	82 *vs* 82	74 *vs* 80	ICSI	GnRH antagonist protocol and r-FSH/hMG	0.5mg leuprolide acetate 6 days after ICSI	n.a.	no placebo	600mg intravaginal micronized progesterone and 1500 IU hCG	3
Razieh, et al., (43)	Iran	90 *vs* 90	90 *vs* 90	ICSI	Long GnRH agonist protocol and r-FSH	0.1mg triptorelin 5 or 6 days after ICSI		placebo	800mg vaginal micronized progesterone	2 or 3
Yildiz, et al., (18)	Turkey	100 *vs* 100 *vs* 100	100 *vs* 84 *vs* 95	ICSI	Long GnRH agonist protocol and r-FSH	1mg leuprolide acetate 6 days after ICSI	two sequential doses 1mg leuprolide acetate 3 and 6 days after ICSI	no placebo	600mg vaginal micronized progesterone, 4mg 17E2	3
Zafardoust, et al., (45)	Iran	50 *vs* 50	43 *vs* 40	ICSI	GnRH antagonist protocol and r-FSH/hMG	0.1mg decapeptil 6 days after ICSI	n.a.	no placebo	800mg vaginal progesterone	3
Benmachiche, et al., (48)	Denmark	165 *vs* 163	165 *vs* 163	IVF/ICSI	GnRH antagonist protocol and r-FSH	0.1mg triptorelin 6 days after ICSI	n.a.	no placebo	4mg E2, 600mg vaginal micronized progesterone, 1500IU hCG	2 or 3
Saharkhiz, et al., (44)	Iran	125 *vs* 125	122 *vs* 118	ICSI	GnRH antagonist protocol and r-FSH	0.1mg triptorelin 6 days after ICSI	n.a.	placebo	400mg vaginal progesterone	2 or 3
Eftekhar, et al., (21)	Iran	84 *vs* 84	84 *vs* 84	IVF/ICSI	GnRH antagonist protocol and r-FSH	n.a.	two sequential doses 1mg leuprolide acetate 3 and 6 days after ICSI	no placebo	progesterone	2 or 3
Fujii, et al., (13)	Japan	309 *vs* 280	309 *vs* 280	IVF/ICSI	Long GnRH agonist protocol and r-FSH	n.a.	continuous 600 μg/d IN buserelin twice daily for 14 days after oocyte retrieval	no placebo	10mg dydrogesterone	2 or 3
Inamdar, et al., (22)	India	213 *vs* 213	213 *vs* 213	IVF	r-hCG	n.a.	three 1 mg doses of lupride 6 days after oocyte retrieval	no placebo	400mg vaginal progesterone, 100mg natural micronized progesterone	2
Pirard, et al., (46)	Belgium	40 *vs* 20	35 *vs* 18	IVF/ICSI	hMG	n.a.	daily administration of 0.25mg buserelin the day before ovulation trigger	no placebo	hMG	3
Qublan, et al., (47)	Jordan	60 *vs* 60	60 *vs* 60	IVF	Long GnRH agonist protocol	n.a.	three 0.1mg triptorelin at 1, 3 and 6 day after oocyte retrieval	placebo	vaginal progesterone	3
Salehpour, et al., (23)	Iran	21 *vs* 23	21 *vs* 23	ICSI	GnRH agonist protocol, hCG	n.a.	daily dose of 0.2mg triptorelin for 10 weeks	no placebo	400mg vaginal progesterone	3
Qu, et al., (19)	China	40 *vs* 40	40 *vs* 40	IVF	r-FSH/hMG	0.1mg decapeptyl 6 days after ICSI	daily injection of 0.1mg decapeptyl for 14 days	n.a.	90mg vaginal progesterone gel, 20mg dydrogesterone tablets	3

LPS, luteal-phase support; SD, single-dose; MD, multiple-dose; ER, embryo transfer; ICSI, intracytoplasmic sperm injection; IVF, in vitro fertilization; GnRH, gonadotropin-releasing hormone; f-FSH, human follicle stimulating hormone; hMG, human menopausal gonadotropin; hCG, human chorionic gonadotropin; n.a., not available.

### 3.3 Quality Assessment of Included Studies

**Figures S1** displayed the results of the Cochrane risk of bias assessment for 16 eligible studies. Only one study (21) was classified as high risk owing to inadequate random sequence generation and allocation concealment. Two studies (19, 42) were classified as high risk because they did not use the correct blinding method. Four studies (18, 42, 45, 46) were classified as high risk owing to high patient attrition or inconsistencies in the amount of attrition between groups. Regarding other bias, two studies (40) were rated having a high risk of bias. Briefly, approximately 13.75% of the studies (18, 19, 21, 40, 42, 45, 46) were classified as high risk of overall bias.

### 3.4 Pairwise Meta-Analysis

We performed several pairwise meta-analyses to evaluate the comparative effects of two protocols with a combined effect size. According to the pooled results, multiple-dose GnRH agonist protocol was associated with increased live birth rate (6 RCTs; 27.42%% *vs* 19.45%; OR 1.80, 95% CI 1.15 to 2.83; *I^2^* = 53%; *p* = 0.01; low-quality evidence; **Figure S2**), higher clinical pregnancy rate (7 RCTs; 37.64%% *vs* 26.73%; OR 1.89, 95% CI 1.01 to 3.56; *I^2^* = 81%; *p* = 0.05; low-quality evidence; **Figure S3**) and lower clinical abortion rate (6 RCTs; 17.43%% *vs* 27.91%; OR 0.55, 95% CI 0.34 to 0.90; *I^2^* = 0%; *p* = 0.02; low-quality evidence; **Figure S5**) compared with control protocol. Single-dose GnRH agonist protocol significantly increased clinical pregnancy rate (9 RCTs; 39.98% *vs* 32.84%; OR 1.45, 95% CI 1.11 to 1.89; *I^2^* = 50.0%; *p* = 0.007; low-quality evidence; **Figure S3**) and multiple pregnancy rate (5 RCTs; 29.97% *vs* 17.91%; OR 2.55, 95% CI 1.12 to 5.78; *I^2^* = 67%; *p* = 0.03; low-quality evidence; **Figure S4**) compared with control protocol. However, multiple-dose GnRH agonist protocol reported higher live birth rate (2 RCTs; 42.74% *vs* 31.43%; OR 0.56, 95% CI 0.26 to 1.19; *I^2^* = 45%; *p* = 0.13; very low-quality evidence; **Figure S2**), clinical pregnancy rate (2 RCTs; 47.58% *vs* 35.0%; OR 0.51, 95% CI 0.20 to 1.31; *I^2^* = 65%; *p* = 0.16; very low-quality evidence; **Figure S3**) and multiple pregnancy rate (2 RCTs; 30.51% *vs* 26.53%; OR 0.69, 95% CI 0.29 to 1.65; *I^2^* = 0%; *p* = 0.40; very low-quality evidence; **Figure S4**) as well as lower clinical abortion rate (2 RCTs; 10.17% *vs* 10.20%; OR 1.11, 95% CI 0.30 to 4.11; *I^2^* = 0%; *p* = 0.88; very low-quality evidence; **Figure S5**) compared with single-dose GnRH agonist protocol although no significant difference was detected.

### 3.5 Network Meta-Analysis

Network meta-analysis based on consistency model further confirmed the efficacious efficacy of multiple-dose GnRH agonist protocol in increasing live birth rate (OR 2.04, 95% CrI 1.19 to 3.93) and clinical pregnancy rate (OR 2.10, 95% CrI 1.26 to 3.54) compared with control protocol (protocol or no protocol). However, single-dose GnRH agonist protocol generated relatively lower point estimates in terms of live birth rate (OR 0.59, 95% CrI 0.27 to 1.11) and clinical pregnancy rate (OR 0.67, 95% CrI 0.36 to 1.24) and relatively higher estimates for multiple pregnancy rate (OR 1.48, 95% CrI 0.39 to 6.40) and clinical abortion rate (OR 1.39, 95% CrI 0.62 to 3.18) compared with multiple-dose GnRH agonist protocol although no statistical difference was detected. Results of network meta-analysis were shown in **Table 2**.

**Table 2:** Results of network meta-analysis.

Outcomes	Comparison, OR (95% CrI)		
	SD *vs* control	MD *vs* control	SD *vs* MD
Live birth rate	1.21 (0.69, 2.03)	**2.04 (1.19, 3.93)**	0.59 (0.27, 1.11)
Clinical pregnancy rate	1.40 (0.89, 2.19)	**2.10 (1.26, 3.54)**	0.67 (0.36, 1.24)
Multiple pregnancy rate	2.15 (0.68, 6.60)	1.45 (0.40, 4.51)	1.48 (0.39, 6.40)
Clinical abortion rate	0.94 (0.55, 1.71)	0.67 (0.36, 1.36)	1.39 (0.62, 3.18)

SD, single-dose; MD, multiple-dose; CrI, creditable interval. Bold numerical value indicates statistical significance.

### 3.6 Consistency Examination

Node-splitting analysis was implemented to evaluate inconsistency by comparing the differences between direct and indirect evidence. For all available comparisons with at least one closed loop, split-node analysis did not suggest inconsistency between direct and indirect evidence, which were summarized in Table S2. As a result, we convinced that results calculated from consistency model were reliable and robust.

### 3.7 Ranking Probability

**Figure 3:**
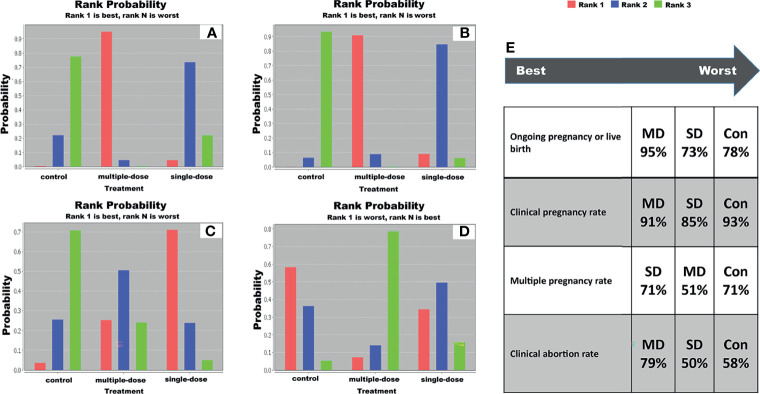
Ranking probability diagram. **(A)** live birth rate, **(B)** clinical pregnancy rate, **(C)** multiple pregnancy rate, **(D)** clinical abortion rate, and **(E)** specific ranking of each protocol in terms of individual outcome. SD, single-dose; MD, multiple-dose; Con, control.

We generated a ranking probability matrix of each outcome using ADDIS software, and results revealed that multiple-dose GnRH agonist protocol was the most effective protocol for all outcomes compared with single-dose GnRH agonist protocol. Specific rank probability of each protocol for available outcomes was summarized in Table S3, and the rank probability diagram was shown in **Figure 3**. According to the results of rank probabilities, multiple-dose GnRH agonist protocol was the best efficacious option for live birth rate (95%), clinical pregnancy rate (91%) and clinical abortion rate (79%), however single-dose GnRH agonist protocol was the best efficacious option for multiple pregnancy rate (71%).

### 3.8 Publication Bias Examination

Owing to the accumulated number of included studies of individual comparison was not more than 10, we therefore did not draw funnel plot to inspect whether the presence of publication bias.

## 4 Discussion

LPD has been identified as a common question in all IVF/ICSI cycles, and various LPS protocols have been routinely used to improve pregnancy outcomes (50). Among available LPS protocols, administration of GnRH agonist protocol introduced recently as a favorable LPS (14). Despite existing, several reports determined the efficacy of single-dose GnRH agonist as a LPS protocol, optimal strategy of GnRH agonist remains undetermined because multiple-dose GnRH agonist protocol commonly used in clinical practice. In this study, we firstly introduced the network meta-analysis to comprehensively investigate the comparative efficacy and safety of single-dose versus multiple-dose GnRH agonist protocols through combining direct and indirect evidence. Based on our results, multiple-dose GnRH agonist protocol was the best efficacious and safe option for increasing the live birth rate and clinical pregnancy rate compared with control protocols. Meanwhile, single-dose GnRH agonist protocol was the best efficacious option for increasing the multiple pregnancy rate although there was no statistical difference between single-dose and multiple-dose GnRH agonist protocols.

It’s noted that GnRH agonist protocol was not routinely utilized as a LPS support in many IVF centers partially resulted from the poor elucidation of the underlying mechanism of GnRH agonist (9, 49). As a result, it’s imperative to deeply optimize the administration strategy of GnRH agonist, such as determination of the optimal dose in the present network meta-analysis, so that the role of GnRH agonist protocol could be definitively clinically investigated during IVF/ICSI cycles. Additionally, safety may be the potential contributor to the application of GnRH agonist protocol (19). Although previously published studies revealed that GnRH agonist protocol might be associated with the increased risk of congenital abnormality, pregnancy loss rates and ectopic pregnancy rates (51, 52), recently published studies further demonstrated the safety of GnRH agonist protocol as LPS protocol (19, 53–55).

Up to now, there were numerous traditional pairwise meta-analyses investigated the efficacy and safety of single-dose GnRH agonist protocol. Among two recent meta-analyses, Song et al. included 8 eligible studies in the final analysis and found that, compared with placebo or no LPS, administration of single-dose GnRH agonist in the luteal-phase significantly increased clinical pregnancy rate and multiple pregnancy rate (16), which was consistent with our findings from pairwise meta-analysis although we included additional one eligible study (44). Moreover, Ma et al. investigated the role of single-dose and multiple-dose GnRH agonist protocols based on subgroup analysis (2), and results suggested that single-dose protocol significantly increased clinical pregnancy rate, ongoing pregnancy rate and live birth rate but not increase multiple pregnancy rate and decrease abortion rate compared with control protocol. Meanwhile, no statistical difference was identified between multiple-dose and control protocol in terms of clinical pregnancy rate. Results from meta-analysis by Ma et al. was partially inconsistent with our findings in pairwise meta-analysis. It’s noted that ongoing pregnancy and live birth were combined as an individual outcome rather than two independent outcomes in our meta-analysis. Moreover, meta-analysis by Ma et al. only included 3 eligible studies to calculate the pooled estimate of the multiple pregnancy rate, however 5 eligible studies were included for this outcome. Therefore, more sample size was accumulated to generate more reliable result in our pairwise meta-analysis. Additionally, Ma et al. also investigated the efficacy of multiple-dose protocol for clinical pregnancy rate based on 4 eligible studies and did not indicate statistical difference. However, our meta-analysis enrolled 7 eligible studies to calculate the pooled estimate and found that multiple-dose GnRH agonist protocol was linked to an increased clinical pregnancy rate. Compared with previous meta-analyses, our meta-analysis has a significant advantage because network meta-analysis technique was introduced to simultaneously combine direct and indirect evidence (56). Our network meta-analysis did not confirm the efficacy of single-dose GnRH agonist protocol for pregnancy outcomes but confirmed multiple-dose GnRH agonist protocol as the best efficacious and safe option due to its advantages in increasing the live birth rate and clinical pregnancy rate.

Our network meta-analysis has several methodological strengths as follow: (a) we applied a comprehensive search strategy to retrieve eligible studies and therefore decrease the risk of publication bias; (b) we firstly incorporated all available data from direct and indirect comparisons to investigate the comparative efficacy and safety of single-dose versus multiple-dose GnRH agonist protocols more precisely; and (c) we calculated rank probabilities to distinguish the differences between single-dose and multiple-dose protocols.

Nevertheless, we recognized some limitations in our network meta-analysis. Firstly, inadequate number of eligible studies for the comparison between multiple-dose and control protocols as well as the comparison of single-dose and multiple-dose protocols was available, which may have adverse impact on our findings. Secondly, different ovarian stimulation protocols were used in eligible studies’; however, subgroup analysis was not performed to differentiate it due to inadequate eligible studies. Thirdly, despite the fact that GnRH agonist protocols were used in all eligible studies, different doses and drugs may introduce heterogeneity across studies. Therefore, our results should also be cautiously interpreted and further comparative study is required to determine the optimal dose and drug. Last but not least, three studies designed multiple-dose protocol, among them, two studies used two sequential doses at 3 and 6 days after ICSI, however another study sued daily injection protocol for 14 days.

## 5 Conclusion

Our network meta-analysis suggested that multiple-dose protocol of GnRH agonist has the significant advantage of higher live birth rate and clinical pregnancy rate than control protocol (placebo or no placebo), and multiple-dose protocol of GnRH agonist has relatively higher point estimates for effects and relatively lower point estimate for safety compared with single-dose protocol of GnRH agonist although no difference was detected. Therefore, multiple-dose protocol of GnRH agonist as the LPS protocol might be the most efficacious and safest option for increasing the live birth rate and clinical pregnancy rate among patients undergoing IVF/ICSI cycles. Moreover, single-dose protocol of GnRH agonist as the LPS protocol might be the most efficacious option for increasing the multiple pregnancy rate. However, as considering limitations of this network meta-analysis, our findings need additional and high-quality RCTs for further confirmation.

## Data Availability Statement

The original contributions presented in the study are included in the article/**Supplementary Material**. Further inquiries can be directed to the corresponding authors.

## Author Contributions

All authors listed have made a substantial, direct, and intellectual contribution to the work and approved it for publication.

## Funding

This work was supported by the Yunnan Ten Thousand Youth Talent Program [(2018)73], the Yunnan Ten Thousand “Famous Doctors” Special Project [ (2019)35], and the Medical Discipline Leader in Health Commission of Yunnan Province [D-2019004]. The sponsors had no role in the design, execution, interpretation, or writing of the study.

## Conflict of Interest

The authors declare that the research was conducted in the absence of any commercial or financial relationships that could be construed as a potential conflict of interest.

## Publisher’s Note

All claims expressed in this article are solely those of the authors and do not necessarily represent those of their affiliated organizations, or those of the publisher, the editors and the reviewers. Any product that may be evaluated in this article, or claim that may be made by its manufacturer, is not guaranteed or endorsed by the publisher.

## Supplementary Material

Supplementary Figure 1 Methodological quality assessment of 16 eligible studies. **(A)** risk of bias summary, and **(B)** risk of bias graph.

Supplementary Figure 2 Meta-analysis of live birth rate. D, single-dose; MD, multiple-dose.

Supplementary Figure 3 Meta-analysis of clinical pregnancy rate. D, single-dose; MD, multiple-dose.

Supplementary Figure 4 Meta-analysis of multiple pregnancy rate. D, single-dose; MD, multiple-dose.

Supplementary Figure 5 Meta-analysis of clinical abortion rate. D, single-dose; MD, multiple-dose.

## References

[1] Banker M Dyer S Chambers GM Ishihara O Kupka M de Mouzon J . International Committee for Monitoring Assisted Reproductive Technologies (ICMART): World Report on Assisted Reproductive Technologies, 2013. Fertil Steril (2021) 116:741–56. doi: 10.1016/j.fertnstert.2021.03.039 33926722

[2] Ma X Du W Hu J Yang Y Zhang X . Effect of Gonadotrophin-Releasing Hormone Agonist Addition for Luteal Support on Pregnancy Outcome *In Vitro* Fertilization/Intracytoplasmic Sperm Injection Cycles: A Meta-Analysis Based on Randomized Controlled Trials. Gynecol Obstet Invest (2020) 85:13–25. doi: 10.1159/000501204 31422404

[3] Fatemi HM . The Luteal Phase After 3 Decades of IVF: What do We Know? Reprod BioMed Online (2009) 19(Suppl 4):4331. doi: 10.1016/S1472-6483(10)61065-6 20034417

[4] van der Linden M Buckingham K Farquhar C Kremer JA Metwally M . Luteal Phase Support for Assisted Reproduction Cycles. Cochrane Database Syst Rev (2015) 2015:Cd009154. doi: 10.1002/14651858.CD009154.pub3 PMC6461197 26148507

[5] Pritts EA Atwood AK . Luteal Phase Support in Infertility Treatment: A Meta-Analysis of the Randomized Trials. Hum Reprod (2002) 17:2287–99. doi: 10.1093/humrep/17.9.2287 12202415

[6] Gizzo S Andrisani A Esposito F Noventa M Di Gangi S Angioni S . Which Luteal Phase Support is Better for Each IVF Stimulation Protocol to Achieve the Highest Pregnancy Rate? A Superiority Randomized Clinical Trial. Gynecol Endocrinol (2014) 30:902–8. doi: 10.3109/09513590.2014.964638 25268567

[7] Wu H Zhang S Lin X Wang S Zhou P . Luteal Phase Support for *In Vitro* Fertilization/Intracytoplasmic Sperm Injection Fresh Cycles: A Systematic Review and Network Meta-Analysis. Reprod Biol Endocrinol (2021) 19:103. doi: 10.1186/s12958-021-00782-5 34229723 PMC8259396

[8] Ciampaglia W Cognigni GE . Clinical Use of Progesterone in Infertility and Assisted Reproduction. Acta Obstet Gynecol Scand (2015) 94(Suppl 161):17–27. doi: 10.1111/aogs.12770 26345161

[9] Pirard C Donnez J Loumaye E . GnRH Agonist as Luteal Phase Support in Assisted Reproduction Technique Cycles: Results of a Pilot Study. Hum Reprod (2006) 21:1894–900. doi: 10.1093/humrep/del072 16556673

[10] Doblinger J Cometti B Trevisan S Griesinger G . Subcutaneous Progesterone Is Effective and Safe for Luteal Phase Support in IVF: An Individual Patient Data Meta-Analysis of the Phase III Trials. PloS One (2016) 11:e0151388. doi: 10.1371/journal.pone.0151388 26991890 PMC4798618

[11] Barbosa MWP Valadares NPB Barbosa ACP Amaral AS Iglesias JR Nastri CO . Oral Dydrogesterone vs. Vaginal Progesterone Capsules for Luteal-Phase Support in Women Undergoing Embryo Transfer: A Systematic Review and Meta-Analysis. JBRA Assist Reprod (2018) 22:148–56. doi: 10.5935/1518-0557.20180018 PMC5982562 29488367

[12] Fatemi HM . Simplifying Luteal Phase Support in Stimulated Assisted Reproduction Cycles. Fertil Steril (2018) 110:1035–6. doi: 10.1016/j.fertnstert.2018.08.019 30396544

[13] Fujii S Sato S Fukui A Kimura H Kasai G Saito Y . Continuous Administration of Gonadotrophin-Releasing Hormone Agonist During the Luteal Phase in IVF. Hum Reprod (2001) 16:1671–5. doi: 10.1093/humrep/16.8.1671 11473961

[14] Tesarik J Hazout A Mendoza C . Enhancement of Embryo Developmental Potential by a Single Administration of GnRH Agonist at the Time of Implantation. Hum Reprod (2004) 19:1176–80. doi: 10.1093/humrep/deh235 15070873

[15] Oliveira JB Baruffi R Petersen CG Mauri AL Cavagna M Franco JG Jr Administration of Single-Dose GnRH Agonist in the Luteal Phase in ICSI Cycles: A Meta-Analysis. Reprod Biol Endocrinol (2010) 8:107. doi: 10.1186/1477-7827-8-107 20825643 PMC2942885

[16] Song M Liu C Hu R Wang F Huo Z . Administration Effects of Single-Dose GnRH Agonist for Luteal Support in Females Undertaking IVF/ICSI Cycles: A Meta-Analysis of Randomized Controlled Trials. Exp Ther Med (2020) 19:786–96. doi: 10.3892/etm.2019.8251 PMC6913329 31885714

[17] Kyrou D Kolibianakis EM Fatemi HM Tarlatzi TB Devroey P Tarlatzis BC . Increased Live Birth Rates With GnRH Agonist Addition for Luteal Support in ICSI/IVF Cycles: A Systematic Review and Meta-Analysis. Hum Reprod Update (2011) 17:734–40. doi: 10.1093/humupd/dmr029 21733980

[18] Yildiz GA Sukur YE Aytac R Ates C . The Addition of Gonadotrophin Releasing Hormone Agonist to Routine Luteal Phase Support in Intracytoplasmic Sperm Injection and Embryo Transfer Cycles: A Randomized Clinical Trial. Eur J Obstetrics Gynecol Reprod Biol (2014) 182:66–70. doi: 10.1016/j.ejogrb.2014.08.026 25238659

[19] Qu D Li Y . Multiple-Dose Versus Single-Dose Gonadotropin-Releasing Hormone Agonist After First *In Vitro* Fertilization Failure Associated With Luteal Phase Deficiency: A Randomized Controlled Trial. J Int Med Res (2020) 48:300060520926026. doi: 10.1177/0300060520926026 32495663 PMC7273566

[20] Fusi FM Brigante CM Zanga L Mignini Renzini M Bosisio C Fadini R . GnRH Agonists to Sustain the Luteal Phase in Antagonist IVF Cycles: A Randomized Prospective Trial. Reprod Biol Endocrinol (2019) 17:103. doi: 10.1186/s12958-019-0543-2 31783862 PMC6884808

[21] Eftekhar M Mirzaei M Mangoli E Mehrolhasani Y . Effects of Multiple Doses of Gonadotropin-Releasing Hormone Agonist on the Luteal-Phase Support in Assisted Reproductive Cycles: A Clinical Trial Study. Int J Reprod BioMed (2021) 19:645–52. doi: 10.18502/ijrm.v19i7.9475 PMC8387711 34458673

[22] Inamdar DB Majumdar A . Evaluation of the Impact of Gonadotropin-Releasing Hormone Agonist as an Adjuvant in Luteal-Phase Support on IVF Outcome. J Hum Reprod Sci (2012) 5:279–84. doi: 10.4103/0974-1208.106341 PMC3604836 23532169

[23] Salehpour S Nazari L Hosseini S Azizi E Borumandnia N Hashemi T . Efficacy of Daily GnRH Agonist for Luteal Phase Support Following GnRH Agonist Triggered ICSI Cycles Versus Conventional Strategy: A Randomized Controlled Trial. JBRA Assist Reprod (2021) 25:368–72. doi: 10.5935/1518-0557.20200077 PMC8312295 33507722

[24] Page MJ Moher D Bossuyt PM Boutron I Hoffmann TC Mulrow CD . PRISMA 2020 Explanation and Elaboration: Updated Guidance and Exemplars for Reporting Systematic Reviews. BMJ (2021) 372:n160. doi: 10.1136/bmj.n160 33781993 PMC8005925

[25] Hutton B Salanti G Caldwell DM Chaimani A Schmid CH Cameron C . The PRISMA Extension Statement for Reporting of Systematic Reviews Incorporating Network Meta-Analyses of Health Care Interventions: Checklist and Explanations. Ann Internal Med (2015) 162:777–84. doi: 10.7326/M14-2385 26030634

[26] Higgins JPT Green S . Cochrane Handbook for Systematic Reviews of Interventions Version 5.1.0 [Updated March 2011]. Chichester: The Cochrane Collaboration (2011). Available at: www.handbook.cochrane.org.

[27] Gordon A Raynes-Greenow C McGeechan K Morris J Jeffery H . Risk Factors for Antepartum Stillbirth and the Influence of Maternal Age in New South Wales Australia: A Population Based Study. BMC Pregnancy Childbirth (2013) 13:12. doi: 10.1186/1471-2393-13-12 23324309 PMC3552834

[28] Reddy UM Laughon SK Sun L Troendle J Willinger M Zhang J . Prepregnancy Risk Factors for Antepartum Stillbirth in the United States. Obstet Gynecol (2010) 116:1119–26. doi: 10.1097/AOG.0b013e3181f903f8 PMC3326407 20966697

[29] Higgins JP Altman DG Gotzsche PC Juni P Moher D Oxman AD . The Cochrane Collaboration's Tool for Assessing Risk of Bias in Randomised Trials. BMJ (2011) 343:d5928. doi: 10.1136/bmj.d5928 22008217 PMC3196245

[30] Bowden J Tierney JF Copas AJ Burdett S . Quantifying, Displaying and Accounting for Heterogeneity in the Meta-Analysis of RCTs Using Standard and Generalised Q Statistics. BMC Med Res Methodol (2011) 11:41. doi: 10.1186/1471-2288-11-41 21473747 PMC3102034

[31] Higgins JP Thompson SG . Quantifying Heterogeneity in a Meta-Analysis. Stat Med (2002) 21:1539–58. doi: 10.1002/sim.1186 12111919

[32] van Valkenhoef G Tervonen T Zwinkels T de Brock B Hillege H . ADDIS: A Decision Support System for Evidence-Based Medicine. Decis Support Syst (2013) 55:459–75. doi: 10.1016/j.dss.2012.10.005

[33] Cipriani A Higgins JPT Geddes JR Salanti G . Conceptual and Technical Challenges in Network Meta-Analysis. Ann Intern Med (2013) 159:130–7. doi: 10.7326/0003-4819-159-2-201307160-00008 23856683

[34] Brooks S Gelman A . General Methods for Monitoring Convergence of Iterative Simulations. J Comput Graphi Stat (1998) 7:434–55. doi: 10.2307/1390675

[35] Burger DA Schall R . A Bayesian Nonlinear Mixed-Effects Regression Model for the Characterization of Early Bactericidal Activity of Tuberculosis Drugs. J Biopharm Stat (2015) 25:1247–71. doi: 10.1080/10543406.2014.971170 PMC4673548 25322214

[36] Dias S Welton NJ Caldwell DM Ades AE . Checking Consistency in Mixed Treatment Comparison Meta-Analysis. Stat Med (2010) 29:932–44. doi: 10.1002/sim.3767 20213715

[37] Albert I Makowski D . Ranking Crop Species Using Mixed Treatment Comparisons. Res Synth Methods (2019) 10:343–59. doi: 10.1002/jrsm.1328 30353974

[38] Singh S Murad MH Chandar AK Bongiorno CM Singal AK Atkinson SR . Comparative Effectiveness of Pharmacological Interventions for Severe Alcoholic Hepatitis: A Systematic Review and Network Meta-Analysis. Gastroenterology (2015) 149:958–70.e12. doi: 10.1053/j.gastro.2015.06.006 26091937

[39] Palma Perez S Delgado Rodriguez M . Practical Considerations on Detection of Publication Bias. Gac Sanit (2006) 20(Suppl 3):10–6. doi: 10.1157/13101085 17433196

[40] Ata B Urman B . Single Dose GnRH Agonist Administration in the Luteal Phase of Assisted Reproduction Cycles: Is the Effect Dependent on the Type of GnRH Analogue Used for Pituitary Suppression? Reprod BioMed Online (2010) 20:165–6. doi: 10.1016/j.rbmo.2009.10.022 20159003

[41] Ata B Yakin K Balaban B Urman B . GnRH Agonist Protocol Administration in the Luteal Phase in ICSI-ET Cycles Stimulated With the Long GnRH Agonist Protocol: A Randomized, Controlled Double Blind Study. Hum Reprod (2008) 23:668–73. doi: 10.1093/humrep/dem421 18192671

[42] Isik AZ Caglar GS Sozen E Akarsu C Tuncay G Ozbicer T . Single-Dose GnRH Agonist Administration in the Luteal Phase of GnRH Antagonist Cycles: A Prospective Randomized Study. Reprod BioMed Online (2009) 19:472–7. doi: 10.1016/j.rbmo.2009.04.001 19909586

[43] Razieh DF Maryam AR Nasim T . Beneficial Effect of Luteal-Phase Gonadotropin-Releasing Hormone Agonist Administration on Implantation Rate After Intracytoplasmic Sperm Injection. Taiwan J Obstet Gynecol (2009) 48:245–8. doi: 10.1016/S1028-4559(09)60297-7 19797013

[44] Saharkhiz N Salehpour S Hosseini S Hosseinirad H Nazari L Saharkhiz N . Effects of Gonadotropin-Releasing Hormone Agonist (GnRH-A) as Luteal Phase Support in Intracytoplasmic Sperm Injection (ICSI) Cycles: A Randomized Controlled Trial. Middle East Fertil Soc J (2020) 25:20. doi: 10.1186/s43043-020-00030-7

[45] Zafardoust S Jeddi-Tehrani M Akhondi MM Sadeghi MR Kamali K Mokhtar S . Effect of Administration of Single Dose GnRH Agonist in Luteal Phase on Outcome of ICSI-ET Cycles in Women With Previous History of IVF/ICSI Failure: A Randomized Controlled Trial. J Reprod Infertil (2015) 16:96–101. 25927026 PMC4386092

[46] Pirard C Loumaye E Laurent P Wyns C . Contribution to More Patient-Friendly ART Treatment: Efficacy of Continuous Low-Dose GnRH Agonist as the Only Luteal Support-Results of a Prospective, Randomized, Comparative Study. Int J Endocrinol (2015) 2015:727569. doi: 10.1155/2015/727569 25945092 PMC4402188

[47] Qublan H Amarin Z Al-Qudah M Diab F Nawasreh M Malkawi S . Luteal Phase Support With GnRH-A Improves Implantation and Pregnancy Rates in IVF Cycles With Endometrium of <or=7 Mm on Day of Egg Retrieval. Hum Fertil (Camb) (2008) 11:43–7. doi: 10.1080/14647270701704768 18320439

[48] Benmachiche A Benbouhedja S Zoghmar A Boularak A Humaidan P . Impact of Mid-Luteal Phase GnRH Agonist Administration on Reproductive Outcomes in GnRH Agonist-Triggered Cycles: A Randomized Controlled Trial. Front Endocrinol (Lausanne) (2017) 8:124. doi: 10.3389/fendo.2017.00124 28663739 PMC5471294

[49] Tesarik J Hazout A Mendoza-Tesarik R Mendoza N Mendoza C . Beneficial Effect of Luteal-Phase GnRH Agonist Administration on Embryo Implantation After ICSI in Both GnRH Agonist- and Antagonist-Treated Ovarian Stimulation Cycles. Hum Reprod (2006) 21:2572–9. doi: 10.1093/humrep/del173 16926261

[50] de Ziegler D Ayoubi JM Frydman R Fanchin R . Luteal Phase Support in Assisted Reproductive Technologies: From Here to There. Fertil Steril (2018) 109:57–8. doi: 10.1016/j.fertnstert.2017.10.031 29307402

[51] Cahill D . The Risks of GnRH Agonist Administration in Early Pregnancy. Ovulation Induction Update ‘98 Parthenon London (1998) 97–106.

[52] Sahin S Ozay A Ergin E Turkgeldi L Kürüm E Ozornek H . The Risk of Ectopic Pregnancy Following GnRH Agonist Triggering Compared With hCG Triggering in GnRH Antagonist IVF Cycles. Arch Gynecol Obstet (2015) 291:185–91. doi: 10.1007/s00404-014-3399-x 25078054

[53] Bar-Hava I Mizrachi Y Karfunkel-Doron D Omer Y Sheena L Carmon N . Intranasal Gonadotropin-Releasing Hormone Agonist (GnRHa) for Luteal-Phase Support Following GnRHa Triggering, a Novel Approach to Avoid Ovarian Hyperstimulation Syndrome in High Responders. Fertil Steril (2016) 106:330–3. doi: 10.1016/j.fertnstert.2016.04.004 27114332

[54] Zhou W Zhuang Y Pan Y Xia F . Effects and Safety of GnRH-A as a Luteal Support in Women Undertaking Assisted Reproductive Technology Procedures: Follow-Up Results for Pregnancy, Delivery, and Neonates. Arch Gynecol Obstet (2017) 295:1269–75. doi: 10.1007/s00404-017-4353-5 28357558

[55] Zhan QT Pan PP Xu XR Lou HY Lou YY Jin F . An Overview of Studies on Psychological Well-Being in Children Born Following Assisted Reproductive Technologies. J Zhejiang Univ Sci B (2013) 14:947–60. doi: 10.1631/jzus.B1300101 PMC3829644 24190441

[56] White IR . Network Meta-Analysis. Stata J (2015) 15:951–85. doi: 10.1177/1536867X1501500403

