# Supplementary figures and images for: Single-Dose Versus Multiple-Dose GnRH Agonist for Luteal-Phase Support in Women Undergoing IVF/ICSI Cycles: A Network Meta-Analysis of Randomized Controlled Trials
Source: Front Endocrinol (Lausanne). 2022 Mar 31;13:802688. doi: 10.3389/fendo.2022.802688 (PMC9008129; doi:10.3389/fendo.2022.802688)

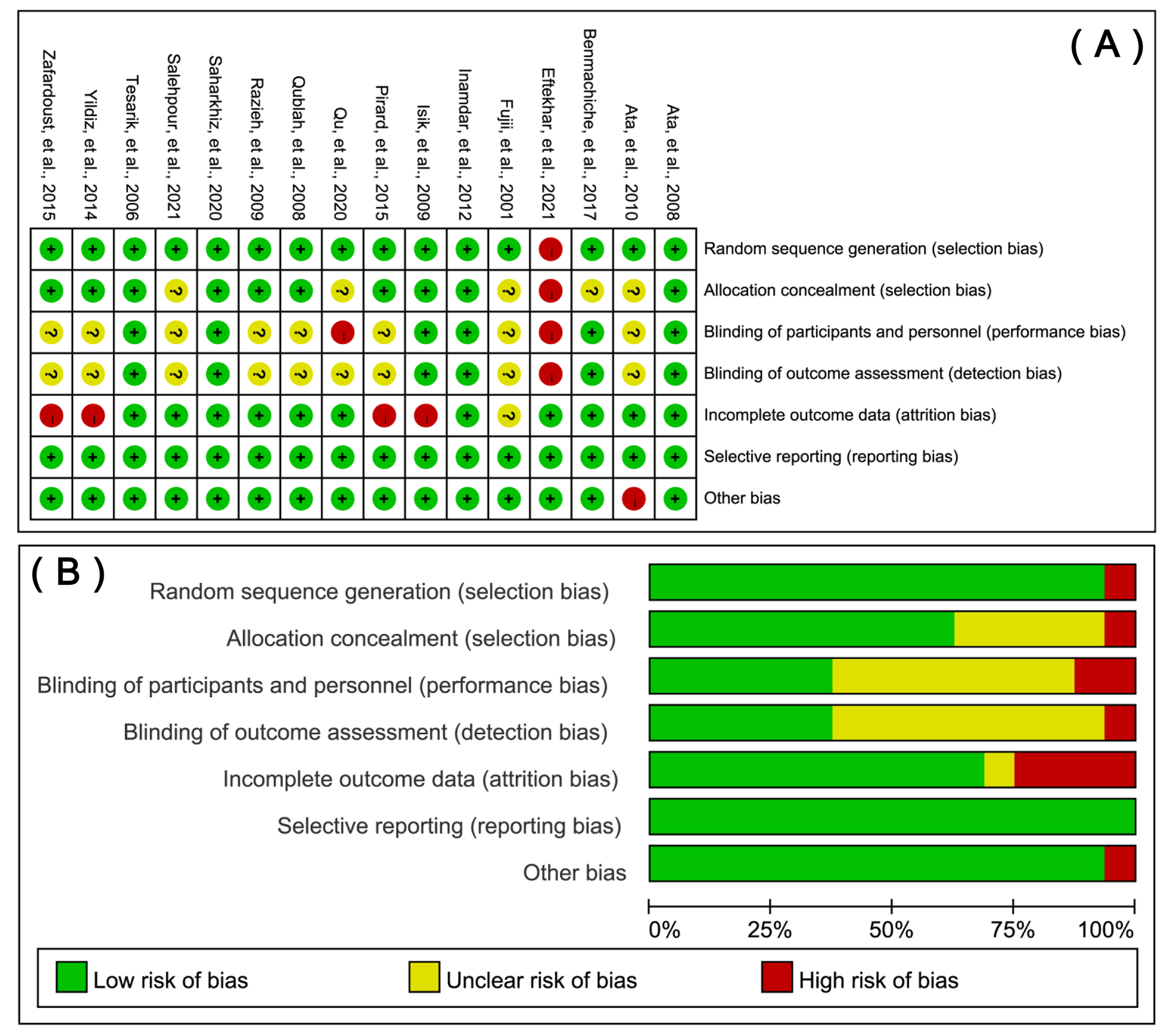

Supplement: Supplementary Figure 1 — Methodological quality assessment of 16 eligible studies. (A) risk of bias summary, and (B) risk of bias graph. [file Image_1.tif]

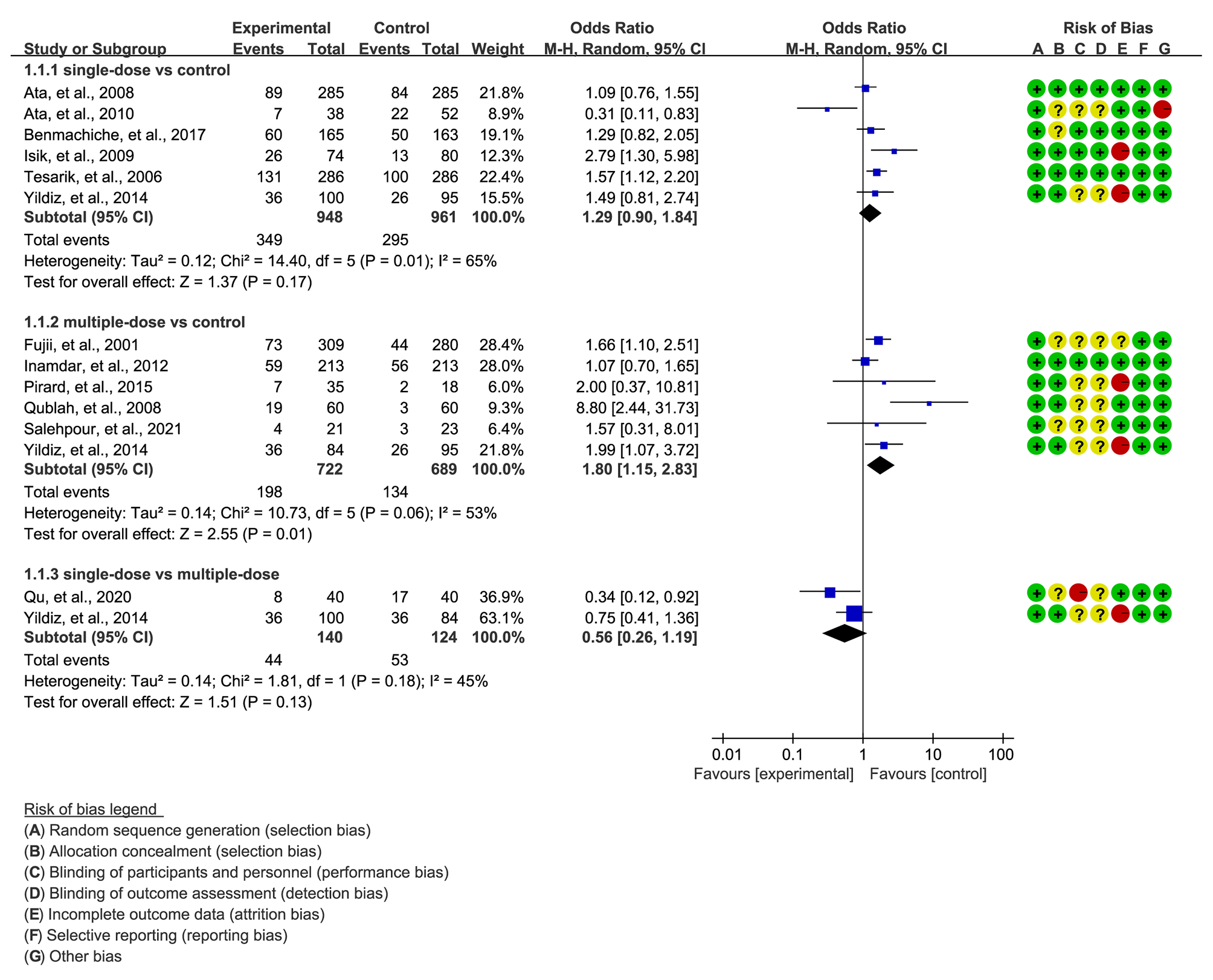

Supplement: Supplementary Figure 2 — Meta-analysis of live birth rate. D, single-dose; MD, multiple-dose. [file Image_2.tif]

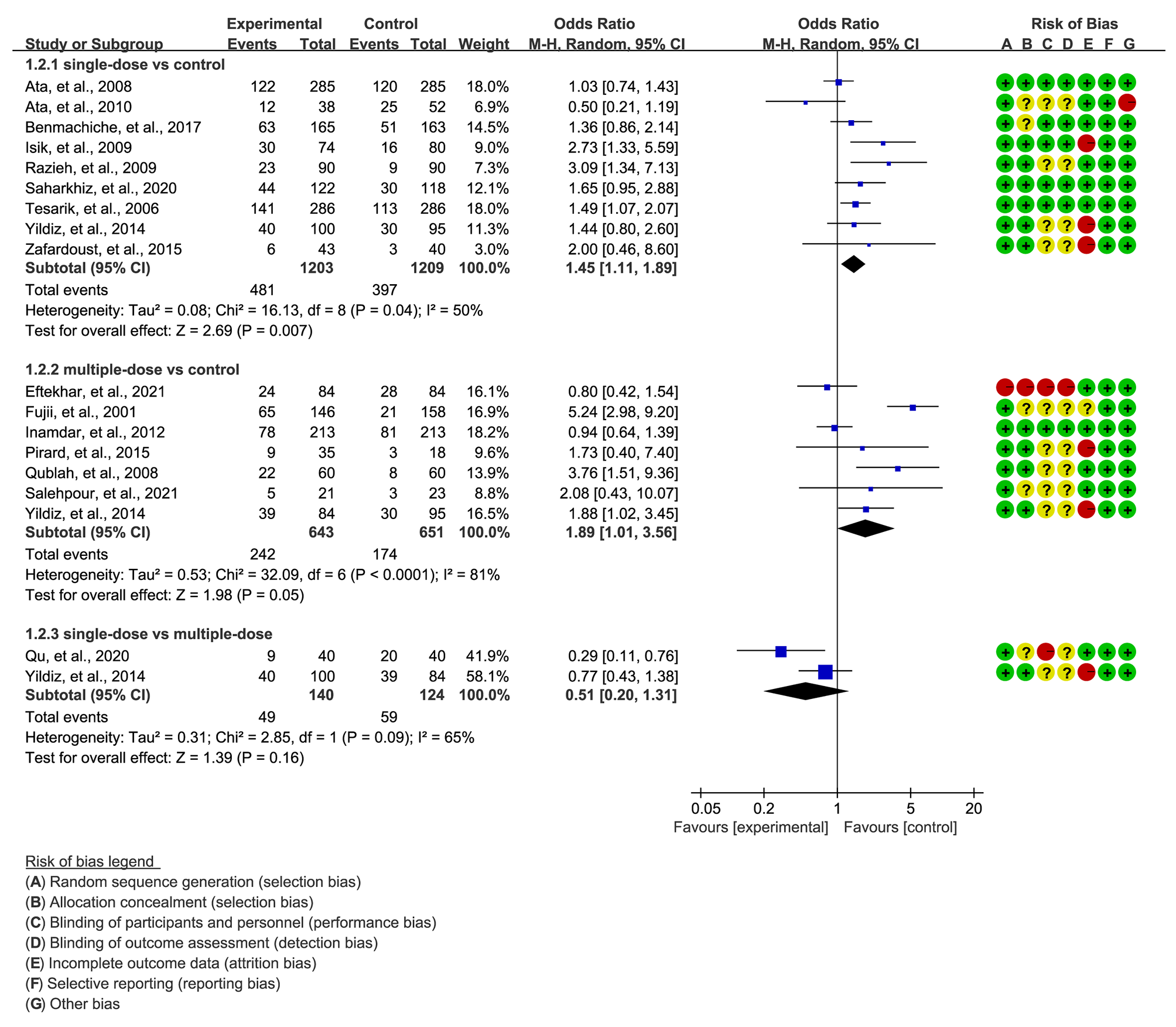

Supplement: Supplementary Figure 3 — Meta-analysis of clinical pregnancy rate. D, single-dose; MD, multiple-dose. [file Image_3.tif]

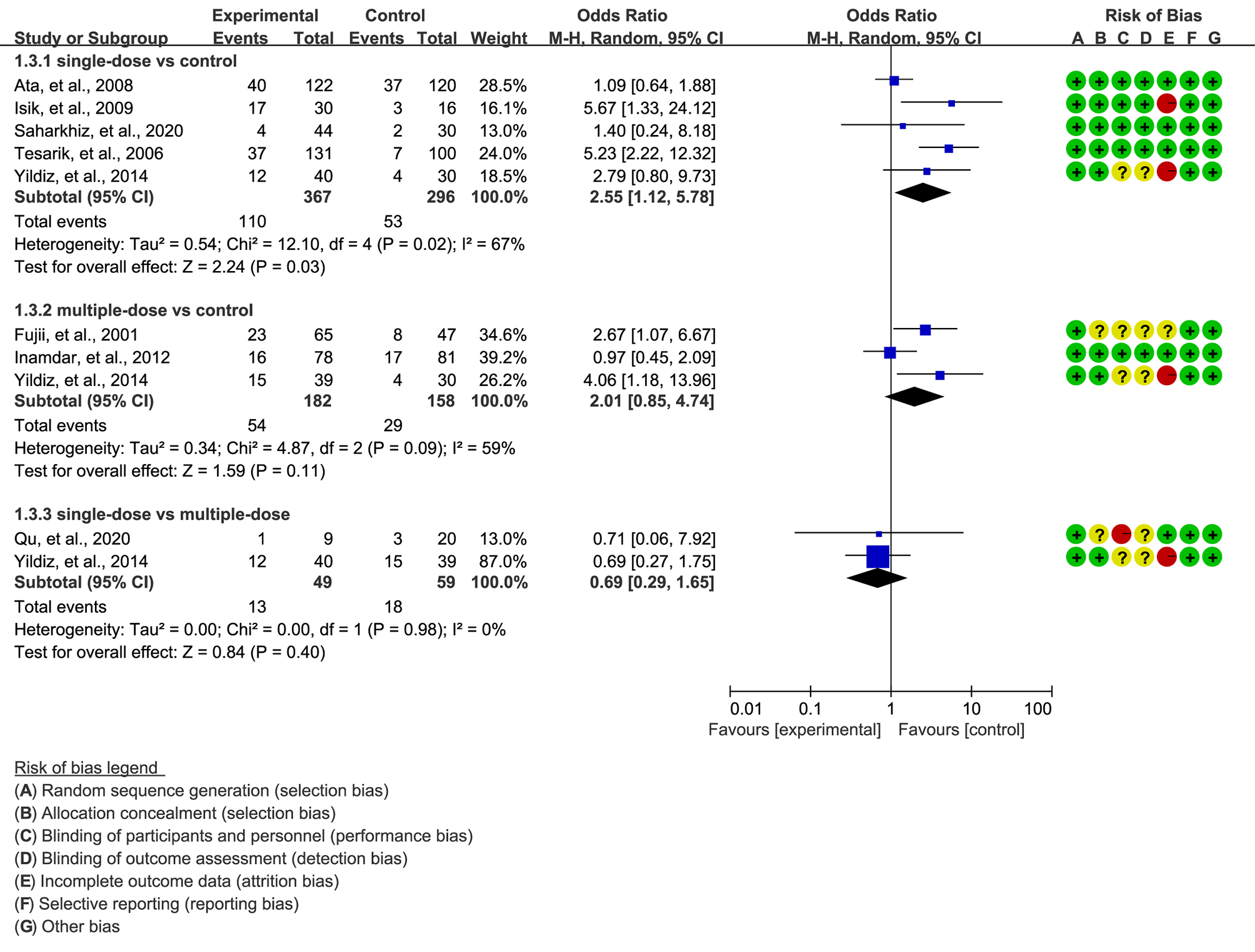

Supplement: Supplementary Figure 4 — Meta-analysis of multiple pregnancy rate. D, single-dose; MD, multiple-dose. [file Image_4.tif]

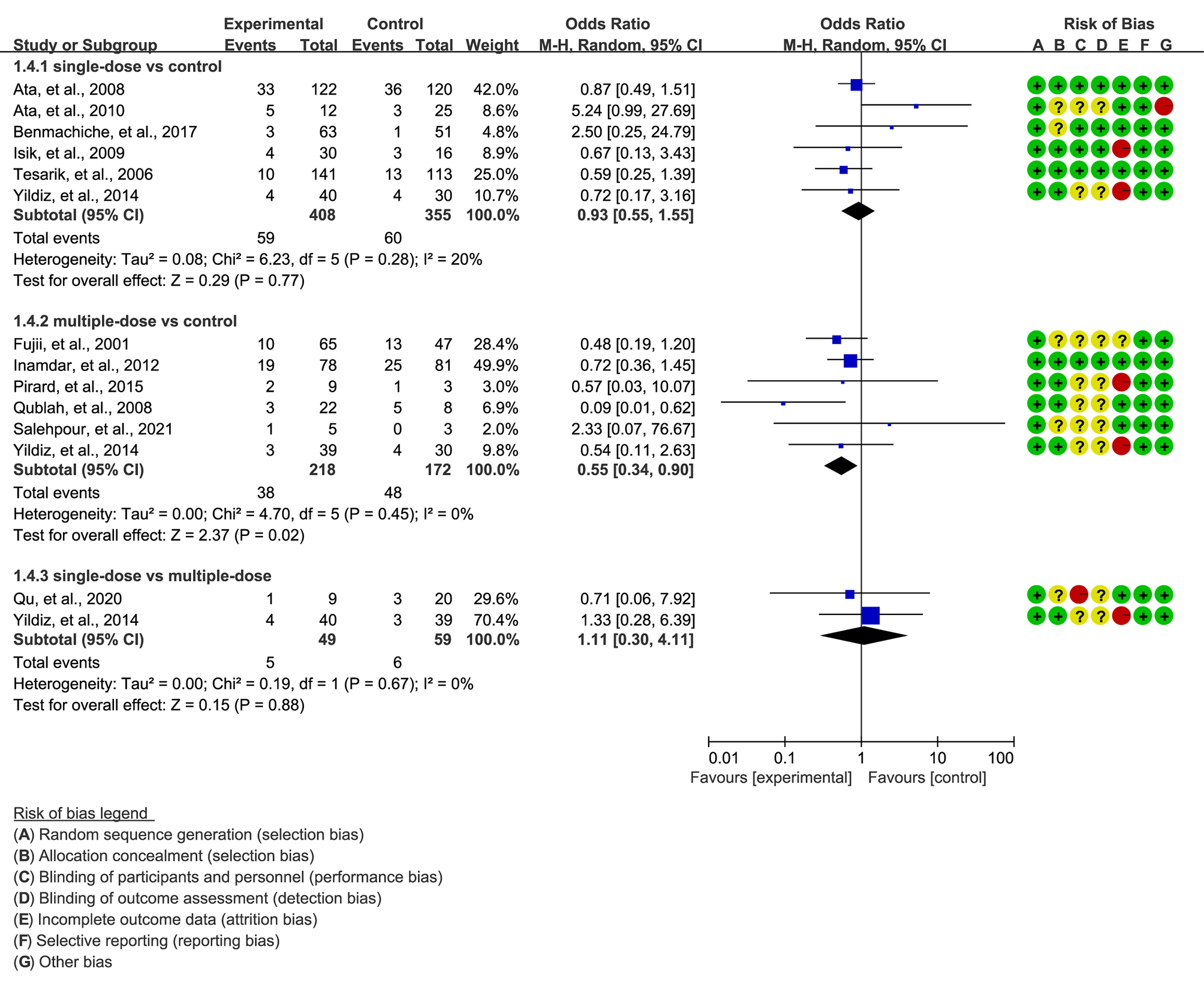

Supplement: Supplementary Figure 5 — Meta-analysis of clinical abortion rate. D, single-dose; MD, multiple-dose. [file Image_5.tif]
