# Supplementary material for: Single-Dose Versus Multiple-Dose GnRH Agonist for Luteal-Phase Support in Women Undergoing IVF/ICSI Cycles: A Network Meta-Analysis of Randomized Controlled Trials
Source: Front Endocrinol (Lausanne). 2022 Mar 31;13:802688. doi: 10.3389/fendo.2022.802688 (PMC9008129; doi:10.3389/fendo.2022.802688)
Supplement: Supplementary file 6 [file Table_1.docx]

**Search strategy of PubMed**

| NO. | Query |
| --- | --- |
| 29 | (((((#11) and (#14 or #17)) and (#20)) and (#27)) not (#28)) |
| 28 | ("Animals"[Mesh]) NOT "Humans"[Mesh] |
| 27 | #21 or #22 or #25 or #26 |
| 26 | (((placebos[Title/Abstract]) OR (placebo*[Title/Abstract])) OR (random*[Title/Abstract])) OR (research design[Title/Abstract]) |
| 25 | #23 and #24 |
| 24 | (mask*[Title/Abstract]) OR (blind*[Title/Abstract]) |
| 23 | (((singl*[Title/Abstract]) OR (doubl*[Title/Abstract])) OR (trebl*[Title/Abstract])) OR (tripl*[Title/Abstract]) |
| 22 | clinical trial[Title/Abstract] |
| 21 | ((((((("Randomized Controlled Trial" [Publication Type]) OR "Randomized Controlled Trials as Topic"[Mesh]) OR "Controlled Clinical Trial" [Publication Type]) OR "Random Allocation"[Mesh]) OR "Double-Blind Method"[Mesh]) OR "Single-Blind Method"[Mesh]) OR "Clinical Trial" [Publication Type]) OR "Clinical Trials as Topic"[Mesh] |
| 20 | #18 or #19 |
| 19 | (((((((((((((((((((((In Vitro Fertilization[Title/Abstract]) OR (In Vitro Fertilizations[Title/Abstract])) OR (Test-Tube Fertilization[Title/Abstract])) OR (Test Tube Fertilization[Title/Abstract])) OR (Test-Tube Fertilizations[Title/Abstract])) OR (Fertilizations in Vitro[Title/Abstract])) OR (Test-Tube Babies[Title/Abstract])) OR (Test Tube Babies[Title/Abstract])) OR (Test-Tube Baby[Title/Abstract])) OR (Embryo Transfers[Title/Abstract])) OR (Blastocyst Transfer[Title/Abstract])) OR (Tubal Embryo Transfer[Title/Abstract])) OR (Tubal Embryo Stage Transfer[Title/Abstract])) OR (Assisted Reproductive Technique[Title/Abstract])) OR (Assisted Reproductive Technics[Title/Abstract])) OR (Assisted Reproductive Technic[Title/Abstract])) OR (Assisted Reproductive Techniques[Title/Abstract])) OR (Assisted Reproductive Technologies[Title/Abstract])) OR (Assisted Reproductive Technology[Title/Abstract])) OR (Intracytoplasmic Sperm Injection[Title/Abstract])) OR (Intracytoplasmic Sperm Injections[Title/Abstract])) OR (ICSI[Title/Abstract]) |
| 18 | ((("Fertilization in Vitro"[Mesh]) OR "Embryo Transfer"[Mesh]) OR "Reproductive Techniques, Assisted"[Mesh]) OR "Sperm Injections, Intracytoplasmic"[Mesh] |
| 17 | #15 or #16 |
| 16 | (multiple[Title/Abstract]) AND (dose[Title/Abstract]) |
| 15 | (multiple-dose[Title/Abstract]) OR (multiple dose[Title/Abstract]) |
| 14 | #12 or #13 |
| 13 | (single[Title/Abstract]) AND (dose[Title/Abstract]) |
| 12 | (single-dose[Title/Abstract]) AND (single dose[Title/Abstract]) |
| 11 | #3 or #10 |
| 10 | #6 and #9 |
| 9 | #7 or #8 |
| 8 | (agonists[Title/Abstract]) OR (agonist[Title/Abstract]) |
| 7 | "agonists" [Subheading] |
| 6 | #4 or #5 |
| 5 | ((((((((((((((((((((((((Gonadotropin Releasing Hormone[Title/Abstract]) OR (Gn-RH[Title/Abstract])) OR (GnRH[Title/Abstract])) OR (Gonadoliberin[Title/Abstract])) OR (LHFSH Releasing Hormone[Title/Abstract])) OR (LH-RH[Title/Abstract])) OR (LHRH[Title/Abstract])) OR (LH-Releasing Hormone[Title/Abstract])) OR (LH Releasing Hormone[Title/Abstract])) OR (LH-FSH Releasing Hormone[Title/Abstract])) OR (LH FSH Releasing Hormone[Title/Abstract])) OR (LHFSHRH[Title/Abstract])) OR (LHRH[Title/Abstract])) OR (Luliberin[Title/Abstract])) OR (Gonadorelin[Title/Abstract])) OR (Luteinizing Hormone-Releasing Hormone[Title/Abstract])) OR (Luteinizing Hormone Releasing Hormone[Title/Abstract])) OR (FSH-Releasing Hormone[Title/Abstract])) OR (FSH Releasing Hormone[Title/Abstract])) OR (Factrel[Title/Abstract])) OR (Cystorelin[Title/Abstract])) OR (Gonadorelin Hydrochloride[Title/Abstract])) OR (Kryptocur[Title/Abstract])) OR (Dirigestran[Title/Abstract])) OR (Gonadorelin Acetate[Title/Abstract]) |
| 4 | "Gonadotropin-Releasing Hormone"[Mesh] |
| 3 | #1 or #2 |
| 2 | ((gonadotropin-releasing hormone agonist[Title/Abstract]) OR (GnRHa[Title/Abstract])) OR (GnRH agonist[Title/Abstract]) |
| 1 | "Gonadotropin-Releasing Hormone/agonists"[Mesh] |

**Search strategy of Embase, which was performed based on OVID platform**

Embase <1974 to 2021 September 27>

NO. Query

1 exp gonadorelin/

2 (Gonadotropin Releasing Hormone or Gn-RH or GnRH or Gonadoliberin or LHFSH Releasing Hormone or LH-RH or LHRH or LH-Releasing Hormone or LH Releasing Hormone or LH-FSH Releasing Hormone or LH FSH Releasing Hormone or LHFSHRH or LHRH or Luliberin or Gonadorelin or Luteinizing Hormone-Releasing Hormone or Luteinizing Hormone Releasing Hormone or FSH-Releasing Hormone or FSH Releasing Hormone or Factrel or Cystorelin or Gonadorelin Hydrochlori or Kryptocurde or Dirigestran or Gonadorelin Acetate).af.

3 1 or 2

4 exp agonist/

5 (agonists or agonist).af.

6 4 or 5

7 3 and 6

8 (gonadotropin-releasing hormone agonist or GnRHa or GnRH agonist).af.

9 7 or 8

10 (single-dose or single dose).af.

11 (single and dose).af.

12 10 or 11

13 (multiple-dose or multiple dose).af.

14 (multiple and dose).af.

15 13 or 14

16 12 or 15

17 exp in vitro fertilization/

18 exp embryo transfer/

19 exp infertility therapy/

20 exp intracytoplasmic sperm injection/

21 17 or 18 or 19 or 20

22 (In Vitro Fertilization or In Vitro Fertilizations or Test-Tube Fertilization or Test Tube Fertilization or Test-Tube Fertilizations or Fertilizations in Vitro or Test-Tube Babies or Test Tube Babies or Test-Tube Baby or Embryo Transfers or Blastocyst Transfer or Tubal Embryo Transfer or Tubal Embryo Stage Transfer or Assisted Reproductive Technique or Assisted Reproductive Technics or Assisted Reproductive Technic or Assisted Reproductive Techniques or Assisted Reproductive Technologies or Assisted Reproductive Technology or Intracytoplasmic Sperm Injection or Intracytoplasmic Sperm Injections or ICSI).af.

23 21 or 22

24 exp randomized controlled trial/

25 exp "randomized controlled trial (topic)"/

26 exp controlled clinical trial/

27 exp randomization/

28 exp double blind procedure/

29 exp single blind procedure/

30 exp clinical trial/

31 exp "clinical trial (topic)"/

32 24 or 25 or 26 or 27 or 28 or 29 or 30 or 31

33 clinical trial.af.

34 (singl* or doubl* or trebl* or tripl*).af.

35 (mask* or blind*).af.

36 34 and 35

37 (placebos or placebo* or random* or research design).af.

38 32 or 33 or 36 or 37

39 exp animal/

40 exp human/

41 39 not 40

42 (9 and 16 and 23 and 38) not 41

43 limit 42 to embase

**Search strategy of the Cochrane registry of controlled trials (CENTRAL), which was conducted based on OVID platform**

| NO. | Query |
| --- | --- |
| 30 | #11 AND #18 AND #21 AND #28 NOT #29 |
| 29 | ("Animals"[Mesh]) NOT "Humans"[Mesh] |
| 28 | #22 OR #23 OR #26 OR #27 |
| 27 | (((placebos[Title/Abstract]) OR (placebo*[Title/Abstract])) OR (random*[Title/Abstract])) OR (research design[Title/Abstract]) |
| 26 | #24 AND #25 |
| 25 | (mask*[Title/Abstract]) OR (blind*[Title/Abstract]) |
| 24 | (((singl*[Title/Abstract]) OR (doubl*[Title/Abstract])) OR (trebl*[Title/Abstract])) OR (tripl*[Title/Abstract]) |
| 23 | clinical trial[Title/Abstract] |
| 22 | ((((((("Randomized Controlled Trial" [Publication Type]) OR "Randomized Controlled Trials as Topic"[Mesh]) OR "Controlled Clinical Trial" [Publication Type]) OR "Random Allocation"[Mesh]) OR "Double-Blind Method"[Mesh]) OR "Single-Blind Method"[Mesh]) OR "Clinical Trial" [Publication Type]) OR "Clinical Trials as Topic"[Mesh] |
| 21 | #19 OR #20 |
| 20 | (((((((((((((((((((((In Vitro Fertilization[Title/Abstract]) OR (In Vitro Fertilizations[Title/Abstract])) OR (Test-Tube Fertilization[Title/Abstract])) OR (Test Tube Fertilization[Title/Abstract])) OR (Test-Tube Fertilizations[Title/Abstract])) OR (Fertilizations in Vitro[Title/Abstract])) OR (Test-Tube Babies[Title/Abstract])) OR (Test Tube Babies[Title/Abstract])) OR (Test-Tube Baby[Title/Abstract])) OR (Embryo Transfers[Title/Abstract])) OR (Blastocyst Transfer[Title/Abstract])) OR (Tubal Embryo Transfer[Title/Abstract])) OR (Tubal Embryo Stage Transfer[Title/Abstract])) OR (Assisted Reproductive Technique[Title/Abstract])) OR (Assisted Reproductive Technics[Title/Abstract])) OR (Assisted Reproductive Technic[Title/Abstract])) OR (Assisted Reproductive Techniques[Title/Abstract])) OR (Assisted Reproductive Technologies[Title/Abstract])) OR (Assisted Reproductive Technology[Title/Abstract])) OR (Intracytoplasmic Sperm Injection[Title/Abstract])) OR (Intracytoplasmic Sperm Injections[Title/Abstract])) OR (ICSI[Title/Abstract]) |
| 19 | ((("Fertilization in Vitro"[Mesh]) OR "Embryo Transfer"[Mesh]) OR "Reproductive Techniques, Assisted"[Mesh]) OR "Sperm Injections, Intracytoplasmic"[Mesh] |
| 18 | #14 OR #17 |
| 17 | #15 OR #16 |
| 16 | (multiple[Title/Abstract]) AND (dose[Title/Abstract]) |
| 15 | (multiple-dose[Title/Abstract]) OR (multiple dose[Title/Abstract]) |
| 14 | #12 OR #13 |
| 13 | (single[Title/Abstract]) AND (dose[Title/Abstract]) |
| 12 | (single-dose[Title/Abstract]) OR (single dose[Title/Abstract]) |
| 11 | #3 OR #10 |
| 10 | #6 AND #9 |
| 9 | #7 OR #8 |
| 8 | (agonists[Title/Abstract]) OR (agonist[Title/Abstract]) |
| 7 | "agonists" [Subheading] |
| 6 | #4 OR #5 |
| 5 | ((((((((((((((((((((((((Gonadotropin Releasing Hormone[Title/Abstract]) OR (Gn-RH[Title/Abstract])) OR (GnRH[Title/Abstract])) OR (Gonadoliberin[Title/Abstract])) OR (LHFSH Releasing Hormone[Title/Abstract])) OR (LH-RH[Title/Abstract])) OR (LHRH[Title/Abstract])) OR (LH-Releasing Hormone[Title/Abstract])) OR (LH Releasing Hormone[Title/Abstract])) OR (LH-FSH Releasing Hormone[Title/Abstract])) OR (LH FSH Releasing Hormone[Title/Abstract])) OR (LHFSHRH[Title/Abstract])) OR (LHRH[Title/Abstract])) OR (Luliberin[Title/Abstract])) OR (Gonadorelin[Title/Abstract])) OR (Luteinizing Hormone-Releasing Hormone[Title/Abstract])) OR (Luteinizing Hormone Releasing Hormone[Title/Abstract])) OR (FSH-Releasing Hormone[Title/Abstract])) OR (FSH Releasing Hormone[Title/Abstract])) OR (Factrel[Title/Abstract])) OR (Cystorelin[Title/Abstract])) OR (Gonadorelin Hydrochloride[Title/Abstract])) OR (Kryptocur[Title/Abstract])) OR (Dirigestran[Title/Abstract])) OR (Gonadorelin Acetate[Title/Abstract]) |
| 4 | "Gonadotropin-Releasing Hormone"[Mesh] |
| 3 | #1 OR #2 |
| 2 | ((gonadotropin-releasing hormone agonist[Title/Abstract]) OR (GnRHa[Title/Abstract])) OR (GnRH agonist[Title/Abstract]) |
| 1 | "Gonadotropin-Releasing Hormone/agonists"[Mesh] |
