# Supplementary material for: Single-Dose Versus Multiple-Dose GnRH Agonist for Luteal-Phase Support in Women Undergoing IVF/ICSI Cycles: A Network Meta-Analysis of Randomized Controlled Trials
Source: Front Endocrinol (Lausanne). 2022 Mar 31;13:802688. doi: 10.3389/fendo.2022.802688 (PMC9008129; doi:10.3389/fendo.2022.802688)
Supplement: Supplementary file 7 [file Table_2.docx]

**Table S2. Consistency examination of available comparison with closed loop.**

| **Comparison** | **Direct effect** | **Indirect effect** | **Overall** | **P-value** |
| --- | --- | --- | --- | --- |
| Live birth rate | | | | |
| Control vs MD | 0.66 (0.06, 1.35) | 1.34 (-0.40, 3.09) | 0.71 (0.17, 1.37) | 0.42 |
| Control vs SD | 0.25 (-0.35, 0.78) | -0.54 (-2.15, 1.26) | 0.19 (-0.36, 0.71) | 0.39 |
| MD vs SD | -0.61 (-1.80, 0.49) | -0.48 (-1.53, 0.41) | -0.53 (-1.32, 0.10) | 0.84 |
| Clinical pregnancy rate | | | | |
| Control vs MD | 0.64 (0.10, 1.21) | 1.68 (0.08, 3.33) | 0.74 (0.23, 1.26) | 0.22 |
| Control vs SD | 0.40 (-0.07, 0.86) | -0.63 (-2.29, 1.01) | 0.33 (-0.12, 0.78) | 0.22 |
| MD vs SD | -0.68 (-1.78, 0.34) | -0.25 (-1.03, 0.55) | -0.40 (-1.03, 0.21) | 0.47 |
| Multiple pregnancy rate | | | | |
| Control vs MD | 0.22 (-1.23, 1.51) | 1.53 (-2.06, 5.74) | 0.37 (-0.92, 1.51) | 0.49 |
| Control vs SD | 0.94 (-0.27, 2.24) | -0.45 (-4.67, 3.26) | 0.77 (-0.38, 1.89) | 0.45 |
| MD vs SD | -0.37 (-2.56, 1.73) | 0.81 (-1.11, 2.91) | 0.40 (-0.95, 1.86) | 0.36 |
| Clinical abortion rate | | | | |
| Control vs MD | -0.45 (-1.16, 0.33) | 0.59 (-2.38, 3.87) | -0.40 (-1.04, 0.30) | 0.51 |
| Control vs SD | -0.06 (-0.60, 0.66) | -1.09 (-4.44, 1.67) | -0.06 (-0.60, 0.54) | 0.47 |
| MD vs SD | 0.05 (-1.42, 1.47) | 0.47 (-0.52, 1.55) | 0.33 (-0.48, 1.16) | 0.64 |

SD, single-dose; MD, multiple-dose.
