# Supplementary material for: Single-Dose Versus Multiple-Dose GnRH Agonist for Luteal-Phase Support in Women Undergoing IVF/ICSI Cycles: A Network Meta-Analysis of Randomized Controlled Trials
Source: Front Endocrinol (Lausanne). 2022 Mar 31;13:802688. doi: 10.3389/fendo.2022.802688 (PMC9008129; doi:10.3389/fendo.2022.802688)
Supplement: Supplementary file 8 [file Table_3.docx]

**Table S3. Rank probability of each protocol for all outcomes.**

| **Protocols** | **Rank 1** | **Rank 2** | **Rank 3** | **Rank 1** | **Rank 2** | **Rank 3** | **Rank 1** | **Rank 2** | **Rank 3** | **Rank 1** | **Rank 2** | **Rank 3** |
| --- | --- | --- | --- | --- | --- | --- | --- | --- | --- | --- | --- | --- |
|  | Live birth rate | | | Clinical pregnancy rate | | | Multiple pregnancy rate | | | Clinical abortion rate | | |
| control | 0.00 | 0.22 | 0.78 | 0.00 | 0.06 | 0.93 | 0.04 | 0.26 | 0.71 | 0.58 | 0.36 | 0.05 |
| multiple-dose | 0.95 | 0.05 | 0.00 | 0.91 | 0.09 | 0.00 | 0.25 | 0.51 | 0.24 | 0.07 | 0.14 | 0.79 |
| single-dose | 0.05 | 0.73 | 0.22 | 0.09 | 0.85 | 0.06 | 0.71 | 0.24 | 0.05 | 0.34 | 0.50 | 0.16 |

For ongoing pregnancy or live birth, clinical pregnancy rate and multiple pregnancy rate, rank 1 was best and rank N was worst. For clinical abortion rate, rank 1 was worst and rank N was best.
